# Supplementary material for: Venetoclax Decreases the Expression of the Spike Protein through Amino Acids Q493 and S494 in SARS-CoV-2
Source: Cells. 2022 Jun 14;11(12):1924. doi: 10.3390/cells11121924 (PMC9221610; doi:10.3390/cells11121924)

Venetoclax Decreases the Expression of the Spike Protein through Amino Acids Q493 and S494 in SARS-CoV-2

Chih-Chieh Chen[1,2], Zhi-Jie Zhuang[1], Chia-Wei Wu[3], Yi-Ling Tan[1], Chen Hsiu Huang[3], Chia-Yi Hsu[4], Eing-Mei Tsai[4,5] and Tsung-Hua Hsieh[3*]

Table S1. Ranking lists of the five times drug screening.

No.	DrugBank ID	Drug name	Structure	Rank list 1	Rank list 2	Rank list 3	Rank list 4	Rank list 5	Average ranking	Clinical indication
1	DB11581	Venetoclax	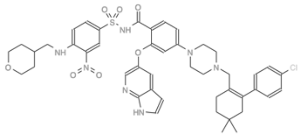	9	1	1	1	1	2.6	Anti-cancer
2	DB09050	Ceftolozane	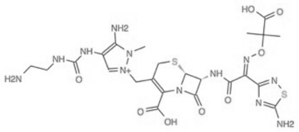	1	7	2	2	2	2.8	Anti-bacterial effect
3	DB09158	Trypan blue free acid	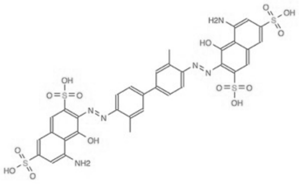	4	5	4	5	8	5.2	Vitrectomy therapy
4	DB00796	Candesartan cilexetil	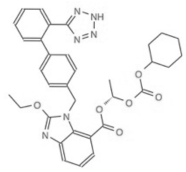	2	17	7	14	3	8.6	Anti-hypertensive
5	DB01072	Atazanavir	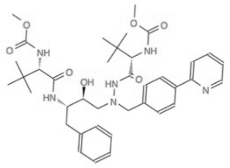	3	20	21	3	6	10.6	Antiretroviral therapy
6	DB01204	Mitoxantrone	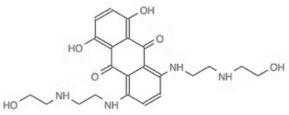	15	27	5	8	4	11.8	Anti-cancer
7	DB06705	Gadofosveset trisodium	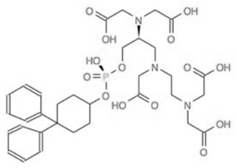	10	10	19	4	24	13.4	Evaluate aortoiliac occlusive disease
8	DB09079	Nintedanib	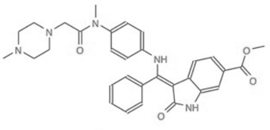	17	28	11	10	13	15.8	Anti-cancer
9	DB08889	Carfilzomib	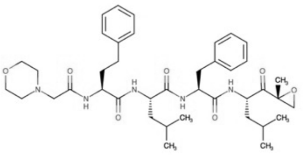	22	19	8	25	20	18.8	Anti-cancer
10	DB01082	Streptomycin	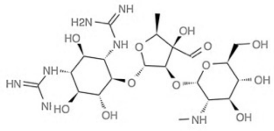	13	18	14	22	42	21.8	Anti-bacterial effect
11	DB11613	Velpatasvir	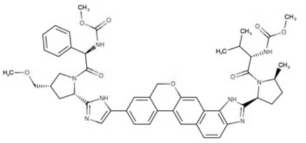	78	2	16	16	7	23.8	Anti-HCV

12	DB01601	Lopinavir	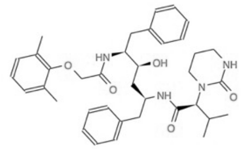	24	30	33	7	33	25.4	Antiretroviral protease inhibitor
13	DB08822	Azilsartan medoxomil	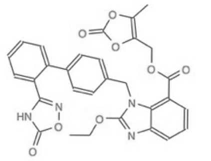	16	9	15	80	11	26.2	Anti-hypertensive
14	DB01421	Paromomycin	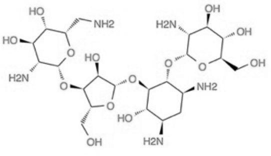	35	13	9	23	53	26.6	Anti-bacterial effect
15	DB11602	Hydroxyethyl cellulose	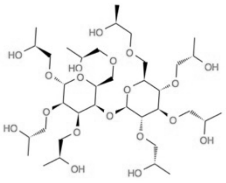	21	12	41	56	14	28.8	Artificial tear solutions
16	DB09297	Paritaprevir	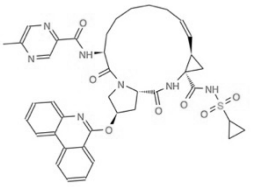	30	57	25	15	18	29	Anti-HCV
17	DB00460	Verteporfin	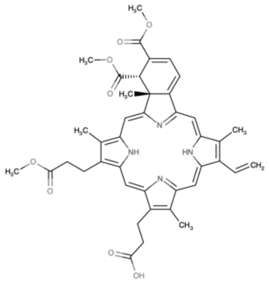	11	38	45	31	27	30.4	Photodynamic therapy
18	DB01232	Saquinavir	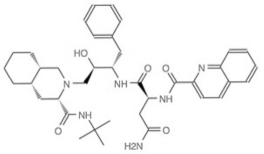	8	76	31	35	9	31.8	Anti-HIV
19	DB00385	Valrubicin	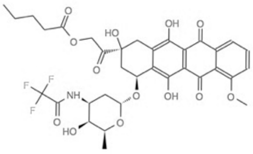	18	14	13	74	50	33.8	Anti-bladder carcinoma
20	DB00284	Acarbose	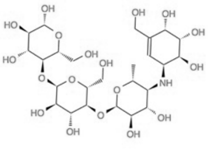	39	4	77	9	47	35.2	Type 2 diabetes mellitus therapy

Full-length gels and blots for2D

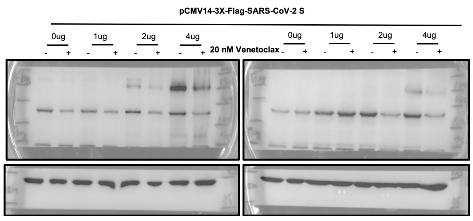

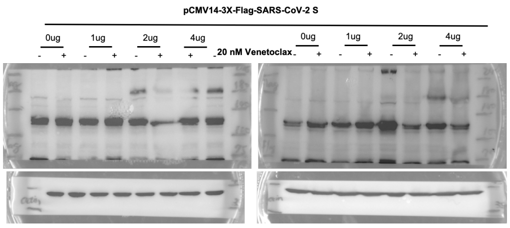

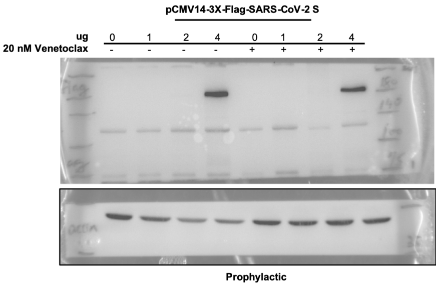

Full-length gels and blots for 2E

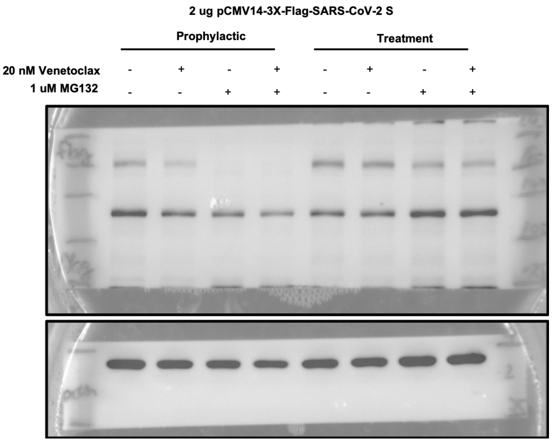

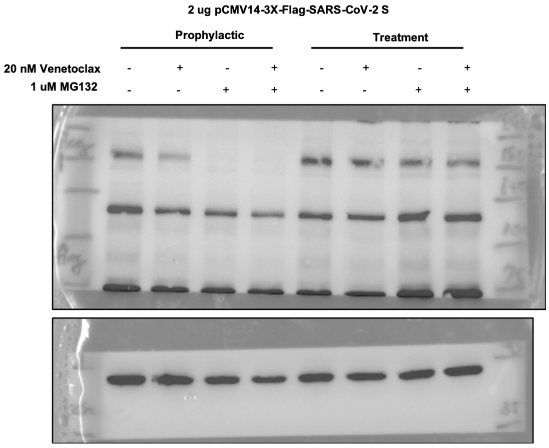

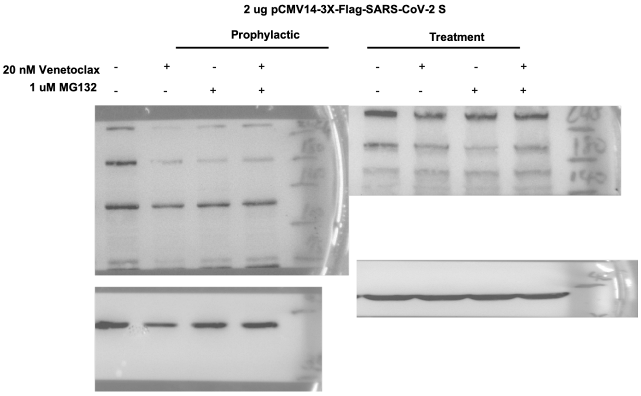

Full-length gels and blots for 3E

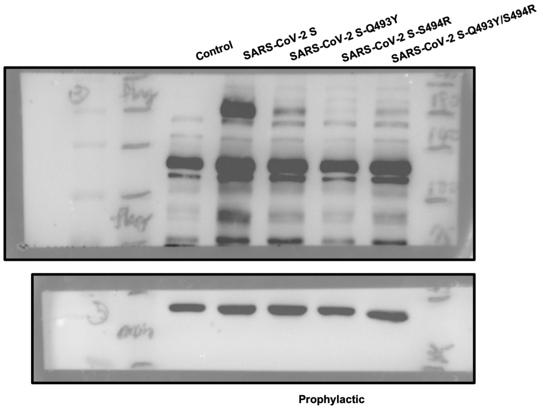

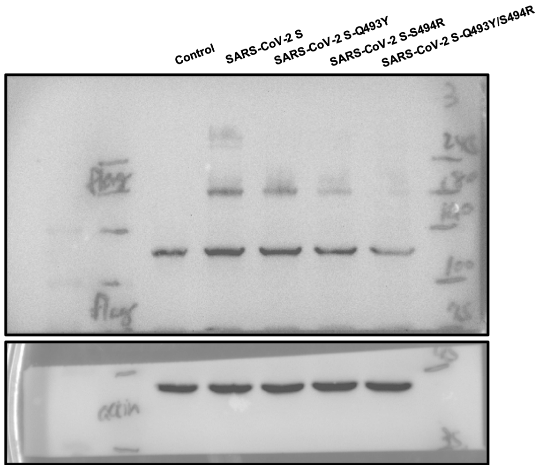

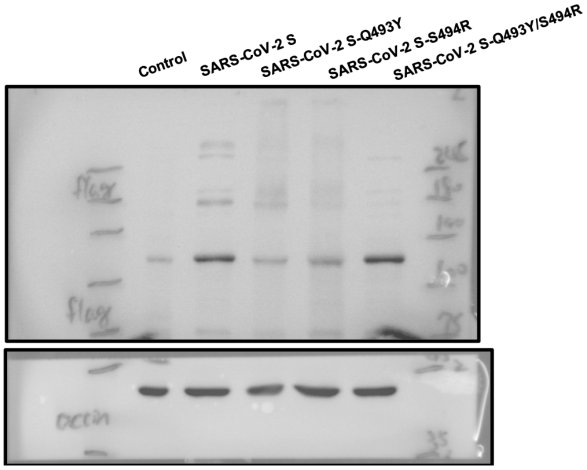

Full-length gels and blots for 3F

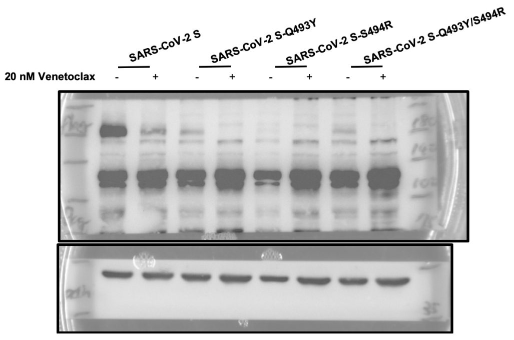

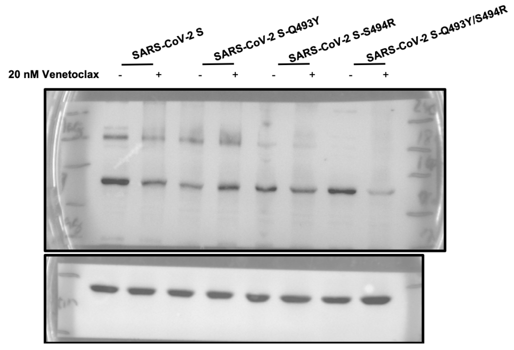

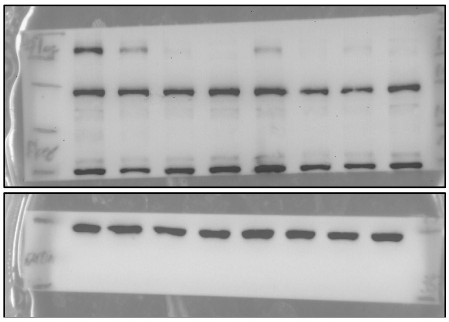

Full-length gels and blots for 3G

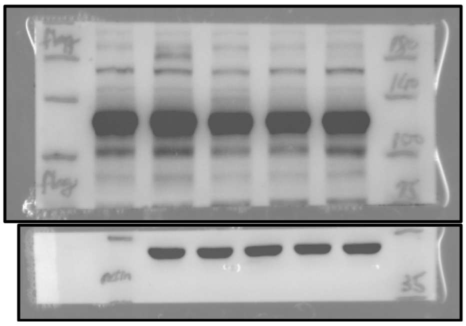

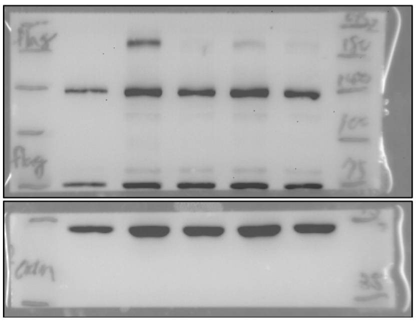

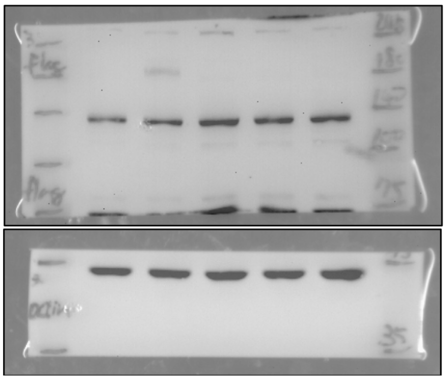

Full-length gels and blots for 3H

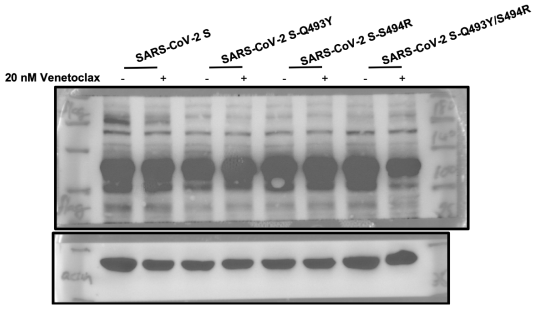

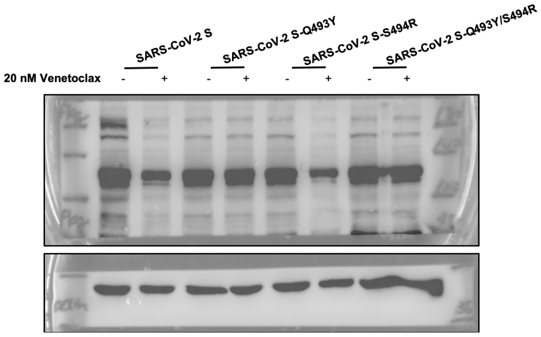

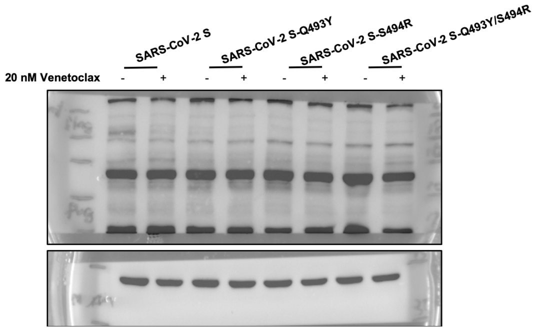

Supplement: Supplementary file 1 [file cells-11-01924-s001.zip › cells-1640323-supplementary.pdf]
